# The Influence of Electroencephalographic Density Spectral Array Guidance of Sevoflurane Administration on Recovery From General Anesthesia in Children. A Randomized Controlled Trial

**DOI:** 10.1111/pan.15065

**Published:** 2025-01-13

**Authors:** Iris J. de Heer, Hannah A. C. Raab, Joost de Vries, Gulhan Karaöz‐Bulut, Frank Weber

**Affiliations:** ^1^ Department of Anesthesia Erasmus University Medical Centre Rotterdam the Netherlands

**Keywords:** electroencephalography, pediatric anesthesia, sevoflurane

## Abstract

**Background:**

In children, monitoring depth of anesthesia is challenging because of the still developing brain. Electroencephalographic density spectral array monitoring provides age‐ and anesthetic drug‐specific electroencephalographic patterns, making it suitable for use in children. Yet, not much is known about the benefits of using density spectral array on post‐operative recovery in children.

**Aim:**

In this randomized controlled trial, the primary aim was to investigate the influence of density spectral array monitoring during general anesthesia on the speed of recovery after surgery.

**Methods:**

Children aged 6 months–12 years scheduled for elective surgery under general anesthesia supplemented with caudal analgesia had either sevoflurane anesthesia titrated to maintain a characteristic density spectral array pattern or based on a predefined end‐tidal sevoflurane concentration of 2.3% (standard care group). The time interval between the discontinuation of sevoflurane and the moment when discharge criteria from the operating room were met (Steward score of 3 or more) was defined as the primary outcome parameter of this trial.

**Results:**

Data from 96 children were analyzed. The time until discharge readiness from the operating room was shorter in group density spectral array (6 min. [13[4–16.8]]) than in group standard care (12 min. [18[6–24.3]]), with a difference between medians of 6 min (95% CI −7 to 0), *p* = 0.041. The mean end‐tidal sevoflurane concentration during the surgical procedure was lower in group density spectral array, 1.8% (0.34) versus 2.3% (0.1) in group standard care (95% CI 0.4–0.7), *p* < 0.001.

**Conclusion:**

This randomized controlled trial provides initial evidence of an added value of density spectral array monitoring in terms of the speed of recovery and allows sevoflurane to be dosed 22% lower during maintenance than with a more traditional approach using a minimal alveolar concentration of 0.9.

**Trial Registration:**

ClinicalTrials.gov identifier: NCT05525104

## 1 Introduction

Depth of hypnosis (DoH) monitoring has many potential benefits, such as appropriate dosage of anesthetic drugs, faster recovery after anesthesia, and preventing adverse events [1, 2]. Most studies on these benefits have been done with index‐based electroencephalographic (EEG) monitors. However, the use of index‐based EEG monitoring in infants or young children is limited because of the algorithms of these index‐based monitors. One of the major limitations of index‐based monitors is the assumption that a single index value, derived from a single algorithm, reflects the same level of unconsciousness for all anesthetics. This assumption is based on the fact that many anesthetics, as long as high doses are used, eventually increase slow oscillations on the EEG [3, 4]. Another common shortcoming of most of these devices is the lack of age‐related EEG expression. Brain maturation during infancy and childhood is an ongoing developmental process, resulting in age‐related EEG features that need to be taken into account to correctly monitor anesthesia depth [5]. In density spectral array (DSA), these limitations are less relevant due to age‐ and anesthesia‐specific EEG expression of the DSA pattern [6, 7, 8, 9, 10, 11]. DSA is a unique two‐dimensional approach to provide all the information of an originally three‐dimensional plot, consisting of the EEG frequency (*y*‐axis), the power of the EEG signal (originally the *z*‐axis, but now color‐coded to be integrated into a 2‐d plot), and the development of the EEG power spectrum over time [11].

Despite the concept of DSA having been published more than half a century ago [12], it is only recently that more has been published about its use in children. Therefore, not much is yet known about the benefits of using DSA in children. To our best knowledge, only one randomized controlled trial has been published on DSA in children. This RCT shows that the use of EEG monitoring (including DSA) during general anesthesia reduces sevoflurane requirements and the incidence of burst suppression [13].

In this randomized controlled trial, we investigate the influence of DSA monitoring during general anesthesia on the speed of recovery after surgery. The primary aim is to determine if DSA‐guided anesthesia (DSA group) will result in faster recovery times from general anesthesia compared to standard care, using a predefined end‐tidal sevoflurane concentration (group standard care). The secondary aims are to determine the difference in total time from discontinuation of anesthetic drug delivery until discharge from the post‐anesthesia care unit, the difference in depth of hypnosis during the procedure, the difference in end‐tidal sevoflurane concentration during the surgical procedure, the difference in intra‐operative blood pressure, the incidence of postoperative delirium, the incidence of recall of events during the procedure (awareness) and the incidence of adverse events.

## 2 Materials and Methods

**FIGURE 1:**
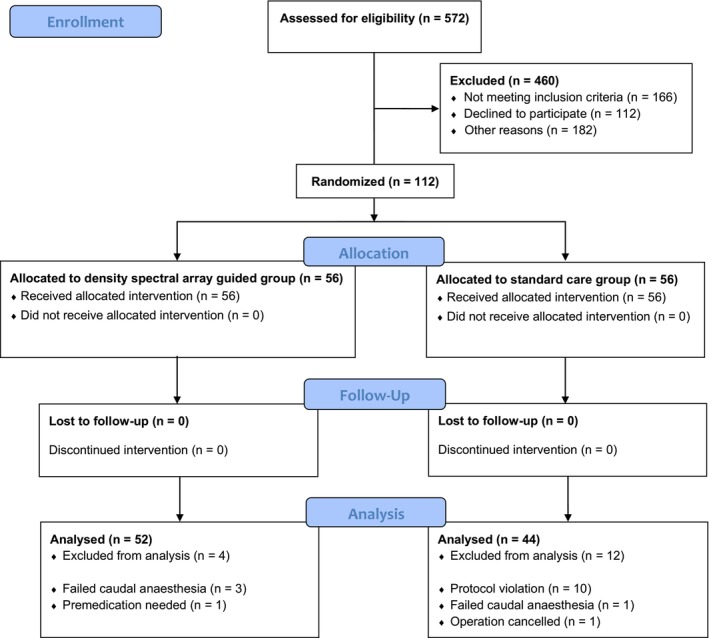
CONSORT diagram of patient recruitment.

This single‐center, prospective, randomized controlled trial was conducted in accordance with the Declaration of Helsinki, approved by the Institutional Review Board of the Erasmus University Medical Center, Rotterdam, the Netherlands (MEC‐2022‐0094; July 28, 2022). Written informed consent was obtained from all the children's parents or legal representatives. This manuscript adheres to the 2010 CONSORT guidelines (see Figure 1).

This study was conducted in the Sophia Children's Hospital, a tertiary children's hospital in Rotterdam, the Netherlands. Patients aged 6 months–12 years scheduled for elective surgery under general anesthesia supplemented with caudal analgesia were eligible for inclusion. To agree to informed consent, the parents or legal guardians had to be able to communicate in Dutch. Primary exclusion criteria were withdrawal of informed consent, (chronic) use of drugs influencing the electroencephalogram such as anti‐epileptics or psychotropic medication, the need for premedication with midazolam or clonidine, and a known intolerance for sevoflurane. Secondary exclusion criteria were protocol violation, failed caudal analgesia, and data registration failure.

### 2.1 Study Procedures

Our institutional research data management system provided us with a computer‐generated block randomization (Castor EDC, provided by the Clinical Trial Center of the Erasmus Medical Centre). Participants were allocated to either the DSA‐guided group (group DSA) or the standard care group (group standard care). The randomization sequence was not accessible to our research team.

The investigator who determined the hypnotic state of the patient, after discontinuation of delivery of the anesthetic drug, was blinded for randomization and was not present at the operating theater during the surgical procedure. At the end of the procedure after discontinuation of the anesthetic drug delivery the Narcotrend monitor was disconnected, turned off and any obscuration had been removed. The investigator entered the theater after that moment, which equals the start of the emergence period. The member of the research team that provided general anesthesia could not be blinded concerning group allocation.

Standard anesthesia monitoring was applied, consisting of an ECG, a non‐invasive blood pressure (NIBP), capnography, and pulse oximetry.

Ventilation was performed with the Dräger Primus anesthesia Workstations (Drägerwerk AG & Co. KGaA, Lübeck, Germany). Electroencephalographic monitoring of the depth of anesthetic drug‐induced hypnosis (DoH) was performed using the Narcotrend EEG monitor (MT Monitortechnik, Bad Bramstedt, Germany). The Narcotrend was originally designed to calculate an index of hypnotic depth, the Narcotrend Index (NI), ranging from 100 (wakefulness) to 0 (very deep hypnosis). Besides this processed index, the Narcotrend monitor also records the EEG power spectrum, relative power in beta (*β*) (%*β*: 13–25 Hz), alpha (*α*) (%*α*: 9–12 Hz), theta (*θ*) (%*θ*: 5–8 Hz) and delta (*δ*) (%*δ*: 1–4 Hz), and DSA. The Narcotrend monitor was attached to the patient's forehead, according to the manufacturer's recommendations, using three electrodes. In patients allocated to the standard care group the screen of the Narcotrend monitor was obscured.

Induction was performed either intravenously (i.v.) with propofol or by inhalation of sevoflurane. After induction, a sufentanil bolus of 0.1 mcg.kg^−1^ i.v. was given before the airway was secured with a supraglottic airway device. Lungs were ventilated with pressure support ventilation modus (PEEP 3 cmH_2_O, end‐tidal CO_2_ target 30–45 mmHg). Caudal analgesia was performed using 1.2 mL.kg^−1^ ropivacaine 0.2%.

In patients allocated to group DSA, directly after the caudal anesthesia was performed, sevoflurane administration was titrated based on the DSA pattern typical for general anesthesia. The DSA pattern to be pursued was defined as consisting of delta and alpha activity, and possibly beta activity [11, 14]. In patients randomized to group standard care, sevoflurane was titrated according to a minimal alveolar concentration (MAC) of 0.9, respectively an end‐tidal sevoflurane concentration of 2.3%, based on standard practice in our pediatric anesthesia department. In group standard care, in case of a clinical suspicion of insufficient DoH (e.g., based on heart rate, blood pressure rise, or patient movement), it was permissible to further increase the sevoflurane concentration. The end‐tidal sevoflurane concentration was checked continuously and recorded every 5 min.

At the end of the procedure, sevoflurane was stopped, and the supraglottic airway device was removed. An investigator blinded to patient group allocation entered the operating room and began to assess the patients' course of recovery from general anesthesia using the “Steward Recovery Score from Anesthesia” [15]. The Steward Recovery Score is available as an Appendix S1.

The time interval between the discontinuation of sevoflurane and the moment when discharge criteria from the operating room are met (Steward score ≥ 3) was defined as the primary outcome parameter of this trial.

The following secondary outcome parameters were defined: the total time from discontinuation of anesthetic drug delivery until discharge from the post anesthesia care unit (Steward score of 6), the differences of depth of hypnosis during the procedure (as measured by the DSA on the Narcotrend monitor), the difference in end‐tidal sevoflurane concentration during the surgical procedure (defined as from start operator until stop operator), the difference in blood pressure drop of more than 2 standard deviations (adjusted for age and gender, based on the reference values for non‐invasive blood pressure in children during anesthesia) [16], the incidence of postoperative delirium by the Cornell Assessment of Postoperative Delirium (CAPD) score [17, 18], the incidence of adverse events for example laryngospasm or bronchospasm, and the incidence of recall of events during the procedure (assessed by a Brice interview [19] on 3 occasions [only performed in patients of at least 6 years of age]). The first interview was assessed on discharge from the recovery room, the second at Day 1 and the third at Day 14.

### 2.2 Power Analysis

We are not aware of any previously published data primarily focused on DSA and the speed of recovery after general anesthesia. A sample size was therefore determined based on clinical experience, expecting a medium to large effect size. We performed a power analysis using the G*power software package with an effect size d of 0.6. The Wilcoxon–Mann–Whitney test was used as a statistical test. A sample size of 51 patients per study group was required to achieve a power of 0.9 with an alpha error probability of 0.05 [20]. To compensate for possible dropouts due to any kind of protocol violation or data registration failure, a sample size of 2× 56 study patients was chosen (+10%).

### 2.3 Statistical Analysis

Statistical analysis was performed using IBM SPSS statistics, version 27.0.1.0 SPSS Inc. and GraphPad Prism [10] for Mac (version 10.0.3) (GraphPad Software Inc., San Diego, CA, USA) for data visualization. Continuous data were tested for normality using the Shapiro–Wilk normality test. Intergroup comparisons of continuous data were performed using an unpaired *t*‐test or Mann–Whitney *U*‐test.

Intergroup DSA signature comparison of hypnotic depth as measured by DSA during surgical procedures was assessed by I.J. de Heer and F. Weber at the end of the study. It was performed as follows: DSA patterns recorded during surgical procedures were categorized and assigned to three conditions of the specific DSA pattern for general anesthesia: continuous appropriate DoH (presence of high power delta, alpha, and possible beta oscillations on the power spectrum or the pattern corrected within 5 min), too deep DoH (presence of high power delta, theta, and alpha oscillations, insufficiently corrected within 5 min or not corrected at all) or too light DoH (presence of high power delta with the absence of high power alpha oscillations and the presence of high power beta oscillations, insufficiently corrected within 5 min or not corrected at all).

Continuous data are presented as mean (sd) or median (95% CI) as appropriate. *p*‐values of < 0.05 were considered significant.

## 3 Results

Between September 2022 and March 2024, 112 pediatric patients were recruited. One patient, who was already randomized to group DSA, asked for premedication with midazolam and was therefore excluded from analysis. Three patients allocated to group DSA, and one in group standard care, were excluded from the analysis because of failed caudal anesthesia. Another secondary exclusion was necessary because the indication for an orchidopexy was found to be incorrect because after induction of anesthesia, both testes were in the scrotum. This patient was randomized to group standard care. During data analysis, we had to exclude another 10 patients allocated to group standard care due to protocol violation. Patient and procedural data of the remaining 96 study participants included in the analysis are presented in Table 1.

**TABLE 1:** Patient and procedure characteristics of patients receiving density spectral array (DSA)‐guided anesthesia or standard care (SC). Data are presented as mean (SD) or absolute values.

	Group DSA, *n* = 52	Group SC, *n* = 44
Age (years)	2.4 (2.7)	2.2 (2.6)
Weight (kg)	13.8 (6.6)	13.6 (6.3)
Gender (Female/Male)	4/48	4/40
ASA score		
1	46	37
2	6	6
3	0	1
Type of surgery		
Orchidopexy	15	18
Inguinal hernia repair	8	9
Urethral valves repair	1	0
Hypospadias correction	13	9
Cystoscopy + sachse or meatotomy	11	3
Other urological procedure	4	4
Orthopedic surgery	0	1
Duration general anesthesia (min.)	69.4 (37.2)	83.7 (39.1)

**FIGURE 2:**
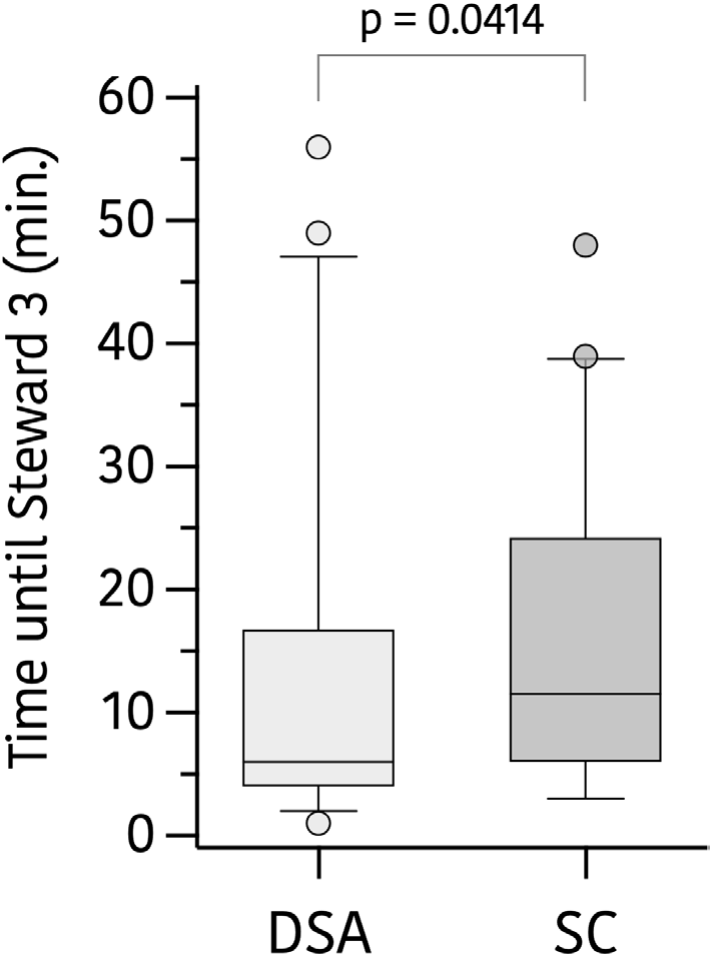
Time interval between the end of the procedure and meeting the criteria for being discharged from the operating room (Steward score of 3) during density spectral array (DSA) guided anesthesia or standard care (SC). Data are medians, with error bars showing interquartile ranges. DSA (light gray), SC (dark gray).

The time interval between the end of the procedure (stop sevoflurane delivery) and meeting the criteria for being discharged from the operating room (Steward score of 3) was shorter in group DSA than in group standard care, with a difference between medians of 6 min (95% CI −7 to 0) minutes, *p* = 0.041, (group DSA 6 min. [13[4–16.8]] versus group SC 12 min. [18[6–24.3]]). For details, see Figure 2.

In total, time from discontinuation of sevoflurane delivery until discharge from the post‐anesthesia care unit (Steward score of 6), no difference was found between both groups, *p* = 0.066 (group DSA 18.5 min. [26[7–32.5]] versus group SC 26.5 min. [28[12.3–40.5]]).

A comparison of the DSA patterns revealed a difference in the DSA pattern during general anesthesia (Table 2).

**TABLE 2:** Comparison of the density spectral array (DSA) patterns during sevoflurane anesthesia.

	Group DSA	Group standard care
DSA pattern representing general anesthesia	46 (88.5%)	24 (54.5%)
DSA pattern representing too deep anesthesia	4 (7.7%)	20 (45.5%)
DSA pattern representing lighter anesthesia	2 (3.8%)	—

The mean end‐tidal sevoflurane concentration during the surgical procedure was lower in group DSA 1.8% (0.34) versus 2.3% (0.1) in the standard care group (95% CI 0.4–0.7), *p* < 0.001.

A post‐operative delirium was not observed in any of the patients. Brice interviews, conducted in all patients over 6 years old, revealed no episodes of awareness with recall during the procedure in any patient. Blood pressure dropped more than 2 SD in three patients, which could be resolved with a fluid bolus of 10 mL.kg^−1^. All of these patients were randomized in group standard care. No other adverse events occurred in any of the participants. One participant had a non‐study‐related serious adverse event, which involved a prolonged admission procedure due to a urological complication.

## 4 Discussion

This randomized controlled trial, which compares recovery times between EEG‐guided anesthesia and standard care anesthesia in children aged 6 months old until 12 years old, provides initial evidence of an added value of density spectral array monitoring in terms of the speed of recovery. This applies to meeting discharge criteria from the operation room (6 vs. 12 min). This could mean operating on one extra patient per day in a busy daycare setting.

It has previously been shown that anesthesia depth monitoring with index‐based EEG monitors ensures faster recovery after anesthesia. When using density spectral array, this was to our best knowledge investigated only once before by Long et al. [13], as a secondary outcome parameter, but no evidence of a difference was found. In that study, a key assumption was that DSA monitoring prevented burst suppression. In our study, a DSA pattern characteristic of general anesthesia, and thus the presence of high‐power delta, alpha, and possible beta oscillations, was pursued in the DSA group, resulting in a significant increase of the speed of recovery.

Intergroup DSA signature comparison revealed a DSA pattern representing deep anesthesia in 20 (45.5%) participants in group SC versus 4 (7.7%) participants in group DSA. This shows that a MAC level of 0.9 or an end‐tidal sevoflurane concentration of 2.3% resulted in deeper anesthesia in 45.5% of the study participants than would have been aimed for under DSA guidance. This underlines that dosing of sevoflurane purely based on MAC value is probably not the best approach.

In 7.7% of participants in group DSA, a DSA pattern was considered too deep during the surgical procedure. This may be due to insufficient rapid washout of the sevoflurane concentration after induction, due to adherence to the principle of low‐flow inhalation anesthesia, resulting in a relatively long period of higher end‐tidal sevoflurane concentrations during the intraoperative phase. Another possible explanation could be our occasional struggles in adjusting the gain of the EEG signal to optimize visualization of the DSA pattern on the Narcotrend monitor.

EEG power increases significantly from infancy to approximately eight years old, and subsequently declines with increasing age [8, 21]. This increase in EEG power with age requires adjustment of the gain of the DoH monitor. Setting the gain correctly optimizes visualization of the DSA spectrum. The Narcotrend monitor does not automatically adjust this gain to the EEG power. The need for manual gain adjustment resulted in occasional struggles to optimize visualization of the DSA pattern on the Narcotrend monitor.

Anesthetic drug overdosage may lead to hemodynamic instability [22] and cause unnecessary anesthetic exposure. However, in the control group, we decided not to reduce the sevoflurane concentration when blood pressure was hypotensive, and instead to stabilize hemodynamics by giving a fluid bolus or using vasopressors if necessary. This decision is because a sevoflurane concentration of ±0.9 MAC is traditionally considered necessary by many (pediatric) anesthesiologists to ensure a sufficient depth of hypnosis [23].

In addition to that, unnecessary anesthetic exposure through a vaporizer, such as that used with sevoflurane, has a negative environmental impact by producing an unnecessary CO_2_ load [8]. In this study, a 22% reduction end‐tidal sevoflurane concentration was seen during the maintenance of anesthesia when DSA monitoring was used. Long et al. recently published a randomized controlled trial about sevoflurane requirements in children during DSA‐guided anesthesia versus standard care. In the study by Long et al., the required end‐tidal sevoflurane concentration during maintenance of anesthesia was 2.23% in the EEG‐guided group versus 2.38% in the standard care group, resulting in a 6.3% difference. The end‐tidal sevoflurane concentration in the standard care group of Long et al. [13] approximately matched the end‐tidal sevoflurane concentration in our standard care group. However, the end‐tidal concentration in the EEG‐guided group was considerably higher than in our study, because in the study of Long et al. sevoflurane was titrated to avoid burst suppression in the EEG‐guided group by maintaining continuous slow‐delta oscillations [13], whereas in our study, sevoflurane was titrated to the most optimal DSA pattern. In the study of Long et al. the EEG‐guided group was primarily guided by the unprocessed EEG, secondarily by the spectrogram, and tertiarily by the patient state index (PSI), to maintain a PSI between 25 and 50.

The relatively large number of secondary exclusions due to protocol violations in the standard care group is considered as a shortcoming of this study. In eight of the secondary‐excluded patients, an end‐tidal sevoflurane concentration of 1.9% was erroneously maintained instead of the targeted concentration of 2.3%. In the remaining two participants who were secondarily excluded, propofol sevoflurane coadministration was administered during maintenance of anesthesia. As a result, the target number of 51 participants per study group was not achieved. Because this exclusion due to protocol violation is completely patient independent, confounding due to exclusion for an outcome‐related reason is not an issue. So, to identify a treatment effect that would occur under optimal conditions, we choose to perform a per‐protocol analysis.

This randomized controlled trial provides initial evidence of an added value of density spectral array monitoring in terms of the speed of recovery. DSA monitoring allows sevoflurane to be dosed 22% lower during maintenance than with a more traditional approach using a minimal alveolar concentration of 0.9.

## 5 Clinical Implications

What is already known: Density spectral array is already used in anesthetic depth monitoring in children, however, not much is known about the benefits of its use.

This study adds new information: It gives initial evidence of an added value of density spectral array monitoring in children regarding the speed of recovery from anesthesia.

## Ethics Statement

This single‐center, prospective randomized controlled trial was conducted in accordance with the Declaration of Helsinki, approved by the Institutional Review Board of the Erasmus University Medical Center, Rotterdam, the Netherlands (MEC‐2022‐0094; July 28, 2022).

## Consent

Written informed consent was obtained from all the children's parents or legal representatives.

## Conflicts of Interest

The authors declare no conflicts of interest.

## Supporting information


Appendix S1.

## Data Availability

The data that support the findings of this study are available on request from the corresponding author, [IdH].
