# Supplementary figures and images for: The Influence of Electroencephalographic Density Spectral Array Guidance of Sevoflurane Administration on Recovery From General Anesthesia in Children. A Randomized Controlled Trial
Source: Paediatr Anaesth. 2025 Jan 13;35(4):287–93. doi: 10.1111/pan.15065 (PMC11883516; doi:10.1111/pan.15065)

VII. Appendices

The Simplified Post-Anesthetic Recovery Score (Steward score) 15.


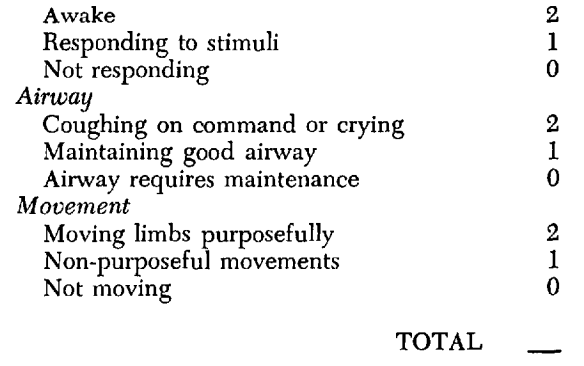

Supplement: Supplementary file 1 — Appendix S1. [file PAN-35-287-s001.docx]
